# Fluoxetine Can Inhibit SARS-CoV-2 In Vitro

**DOI:** 10.3390/microorganisms9020339

**Published:** 2021-02-09

**Authors:** Arthur Dechaumes, Magloire Pandoua Nekoua, Sandrine Belouzard, Famara Sane, Ilka Engelmann, Jean Dubuisson, Enagnon Kazali Alidjinou, Didier Hober

**Affiliations:** 1 Laboratoire de Virologie ULR3610, Univ Lille, CHU Lille, 59000 Lille, France; a.dechaumes@gmail.com (A.D.); magloire.nekoua@gmail.com (M.P.N.); famara.sane@chru-lille.fr (F.S.); ilka.engelmann@chru-lille.fr (I.E.); enagnonkazali.alidjinou@chru-lille.fr (E.K.A.); 2 Virologie Moléculaire et Cellulaire des Coronavirus, Centre D’infection et D’immunité de Lille, Institut Pasteur de Lille, Université de Lille, CNRS, Inserm, CHRU, 59000 Lille, France; sandrine.belouzard@ibl.cnrs.fr (S.B.); jean.dubuisson@ibl.cnrs.fr (J.D.)

**Keywords:** SARS-CoV-2, coronavirus, fluoxetine, in vitro

## Abstract

An outbreak of severe acute respiratory syndrome coronavirus 2 (SARS-CoV-2) resulted in the coronavirus disease pandemic, drastically affecting global health and economy. Though the understanding of the disease has improved, fighting the virus remains challenging. One of the strategies is repurposing existing drugs as inhibitors of SARS-CoV-2. Fluoxetine (FLX), a selective serotonin reuptake inhibitor, reportedly inhibits the replication of RNA viruses, especially Coxsackieviruses B (CVB), such as CV-B4 in vitro and in vivo. Therefore, in this study, we investigated the in vitro antiviral activity of FLX against SARS-CoV-2 in a model of acute infection. When 10 μM of FLX was added to SARS-CoV-2-infected Vero E6 cells, the virus-induced cytopathic effect was not observed. In this model, the level of infectious particles in the supernatant was lower than that in controls. The level was below the limit of detection of the assay up to day 3 post-infection when FLX was administered before viral inoculation or simultaneously followed by daily inoculation. In conclusion, FLX can inhibit SARS-CoV-2 in vitro. Further studies are needed to investigate the potential value of FLX to combat SARS-CoV-2 infections, treat SARS-CoV-2-induced diseases, and explain the antiviral mechanism of this molecule to pave way for novel treatment strategies.

## 1. Introduction

The city of Wuhan, China, first faced the outbreak caused by severe acute respiratory syndrome coronavirus 2 (SARS-CoV-2) in December 2019. The World Health Organization declared the epidemic to be a public health emergency of international concern, and the outbreak quickly grew into a global pandemic. Most common symptoms of COVID-19 include fever, cough, dyspnea, and myalgia. The most common complication is acute respiratory distress syndrome, affecting 3.4% of infected patients and 15.6–17.0% of severe patients [1]. In January 2021, a cumulative total of nearly 97 million confirmed cases and 2 million deaths have been reported since the start of the outbreak (WHO 2021).

The survival of SARS-CoV-2 in the environment (air, surfaces, and water) depends on the size of particulate matter and aerosols, humidity, and temperature [2]. Since environmental pollutants have been shown to alter the immune system, several studies showed that air pollution significantly increases the risk of acute respiratory infection and the severity of COVID-19 [3,4,5,6]. It was also reported that emission of immunomodulating bioactive volatile organic compounds (VOCs) by evergreen forested areas has a protective role on population against SARS-CoV-2 which suggests the importance of nature conservation in reducing the harmful effects of the global pandemic [7].

SARS-CoV-2 is a positive sense RNA virus with a relatively large genome of around 30 kb belonging to the Coronaviridae family. Its genome encompasses five major open reading frames, namely replicase polyproteins, nucleocapsid proteins, spike proteins, envelope proteins, and membrane glycoproteins [8], with a 5′-methylguanosine cap at the beginning and a 3′-poly-A tail at the end encompassing 6 to 10 genes. The order of the genes is usually highly conserved; the first gene is replication and transcription-related and the rest, structural. The replication and transcription-related gene is transcribed and then translated into two large non-structural polyproteins [9]. The spike protein, envelope protein, membrane protein, nucleocapsid protein, 3CL protease, papain-like protease, RNA polymerase, and helicase protein have been suggested as viable antiviral drug targets [10].

Though trials on SARS-CoV-2 genome-based vaccines and therapeutic antibodies are being tested, they represent a long-term solution and short-term solutions such as standard treatments against SARS-CoV-2 are lacking; the wide range of antivirals currently being marketed show no significant activity. Therefore, repurposing existing drugs and antiviral agents is pragmatic and an efficient short-term approach; however, it remains crucial to determine new potential antiviral targets. Several antivirals (remdesivir, favipiravir) and antimalarials (chloroquine, hydroxychloroquine) have emerged as potential therapies [11,12,13]. Despite the candidate drugs such as remdesivir and hydroxychloroquine target different viral infection response pathways or directly interfere with the SARS-CoV-2 replication cycle in vitro, none of them revealed any effect on mortality, mechanical ventilation, and length of hospital stay of patients with COVID-19 [14,15].

Analogous to coronaviruses, Picornaviridae family members such as coxsackieviruses B (*Enterovirus* genus) are also positive sense RNA viruses but with smaller genome size (around 7.5 kb) [16]. Multiple direct-acting inhibitors, such as capsid binders or inhibitors of viral enzymes required for genome replication, have been clinically evaluated, but some of them failed because of limited efficacy or toxicity issues [17]. Hence, host-targeting inhibitors with potential broad-spectrum activity should be a useful strategy to fight enterovirus and coronavirus infections.

Recently, fluoxetine (FLX), a selective serotonin reuptake inhibitor, has been reported as an inhibitor of several viruses, such as dengue [18], hepatitis C [19], SARS-CoV-2 [20,21], and enterovirus in vitro [22].

This drug was formerly used to treat depression and other mental disorders. We and others previously demonstrated a significant antiviral activity against coxsackieviruses B (CVB) 1–4 in vivo and in vitro [23,24,25,26]. FLX can inhibit the replication of CVB4 E2 in a cell line (Panc-1) model of human pancreatic cells [25]. Owing to these inhibitory characteristics of FLX in vitro and in vivo, we investigated the antiviral activity of FLX against SARS-CoV-2 in Vero E6 cells.

## 2. Materials and Methods

SARS-CoV-2 was isolated from a positive respiratory specimen tested using the polymerase chain reaction (PCR) test at the Department of Virology, CHU Lille. VERO-81 cells were transduced with a retroviral vector expressing TMPRSS2 to enhance virus entry [27]. Cells were seeded in T125 flasks and inoculated with 500 µL of the oropharyngeal specimen. Cytopathic effects (CPE) were observed 48 h later, and supernatants were collected 72 h after inoculation. TMPRSS2-transduced VERO-81 cells were subjected to 100 µL of these supernatants. Then, these supernatants were collected following the appearance of CPEs. The isolate was amplified in TMPRSS2-transduced VERO-81 cells and passaged twice to produce the viral stock. Viral stocks were stored in liquid nitrogen (−196 °C) with no stabilizer. After thawing, they were inoculated in the medium containing Vero E6 cells. For virus propagation, SARS-CoV-2 was cultured in Vero E6 cells (ATCC) maintained in DMEM (Gibco) supplemented with 10% heat-inactivated fetal bovine serum (Gibco), 100 U/mL penicillin, and 100 μg/mL streptomycin (Gibco) in T75 flasks and incubated at 37 °C/5% CO_2_. The specificity of the SARS-CoV-2 isolate was assessed using real-time reverse transcription (qRT)-PCR. Viral RNA extraction was performed with the MagNA Pure Compact Nucleic Acid Isolation Kit using the MagNA Pure Compact Instrument (Roche Life Science, Meylan, France). SARS-CoV-2 detection was performed with an in-house real-time duplex RT-PCR assay, targeting two regions in the RdRp gene (IP2 and IP4), designed by the Pasteur Institute of Paris [28]. GAPDH was used as the housekeeping gene to monitor specimen quality, extraction, and PCR inhibition.

The CV-B4 E2 strain was provided by Ji-Won Yoon (Julia McFarlane Diabetes Research Centre, Calgary, AB, Canada). This strain was propagated in HEp-2 cells (ATCC) that were cultivated in complete MEM medium (Gibco) enriched with 10% heat-inactivated fetal bovine serum. Culture supernatants were recovered 5 days post-infection, centrifuged at 800× *g* for 5 min at 20 °C, and filtered through 0.22-µm filter membranes. Aliquots were stored at –80 °C. The virus titers were determined as the tissue culture 50% infectious dose TCID_50_/mL using the Spearman–Karber method.

Fluoxetine hydrochloride (Sigma-Aldrich, Saint-Quentin Fallavier, France) was dissolved in dimethyl sulfoxide (DMSO). To evaluate the antiviral activity of FLX, Vero E6 cells were seeded in 24-well plates at 5 × 10^4^ cells per well and inoculated with the virus at a MOI of 0.01 mixed with various dilutions of FLX or DMSO. The plates were incubated at 37 °C, and cell cultures were observed for CPE daily.

CPE was observed under an inverted microscope (Magnification ×100). Orangu^TM^ (Cell Guidance Systems, Cambridge, UK) was used to measure cell viability according to the manufacturer’s instructions. Viral titers determined in supernatants of infected cells were assessed using the endpoint dilution assay, and the Spearman–Karber statistical method was used to determine the tissue culture 50% infectious dose (TCID_50_/mL).

## 3. Results

A SARS-CoV-2 isolate obtained from a respiratory sample was able to infect a Vero E6 cell monolayer in less than 3 days with a high titer at 10^7.5^ TCID_50_/mL. We studied this in vitro model of SARS-CoV-2 infection to evaluate the antiviral activity of FLX against the virus.

**Figure 1:**
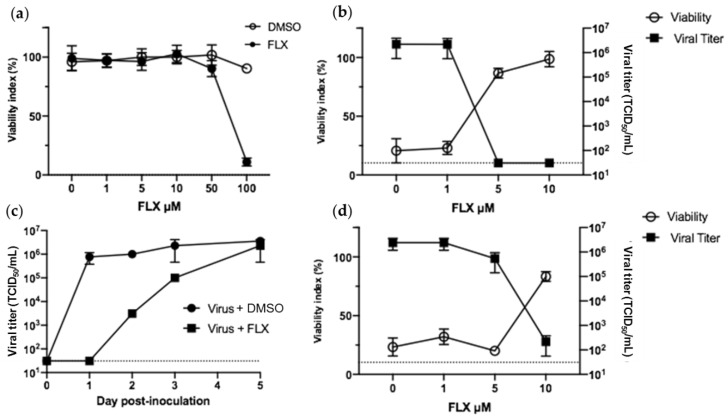
Fluoxetine (FLX) can inhibit the replication of severe acute respiratory syndrome coronavirus 2 (SARS-CoV-2) and CV-B4 E2 in Vero E6 cells. Vero E6 cells were seeded in 24-well plates at 10^5^ cells per well. (**a**) DMSO or various concentrations of FLX dissolved in DMSO were added to cell cultures to determine the cytotoxic concentrations. Cell viability was assessed using the Orangu assay after 72 h. Optical density values are normalized using the viability value of uninfected cells (mock = 100%). (**b**) Vero E6 cells were inoculated with CV-B4 E2 at MOI 0.01 and various concentrations of FLX dissolved in DMSO were added to cell cultures at non-cytotoxic concentrations; the cells were washed 2 h post-inoculation (DMSO conditions not shown). (**c**) Cell cultures were inoculated with SARS-CoV-2 at MOI 0.01 and 10 μM of FLX; cells were washed 2 h post-inoculation. (**d**) Vero E6 cells were inoculated with SARS-CoV-2 at MOI 0.01 and various concentrations of FLX dissolved in DMSO at non-cytotoxic concentrations. The cells were washed 2 h post-inoculation, and either FLX was added at the same concentration or DMSO (DMSO conditions not shown). Day 2 post-infection, cell viability was expressed as % compared with uninfected FLX-treated cells and levels of infectious particles were determined using the endpoint dilution assay. The Spearman–Karber method was used to determine the tissue culture 50% infectious dose (TCID_50_/mL). The results are expressed as the mean ± SD of three independent experiments.

FLX concentrations beyond 50 μM reduced the viability of Vero E6 (Figure 1a). Therefore, concentrations ranging from 0 to 10 μM were used to evaluate the antiviral activity of FLX in these cells. As it was reported that FLX can inhibit the infection of HEp-2 cells with CV-B4, we decided to first study whether FLX can inhibit the infection of Vero E6 cells with CV-B4; 10 μM of FLX was added to Vero E6 cells cultures along with CV-B4 E2, the cells were incubated for 2 h, and then cultures were washed before continuing with incubation. The infection was inhibited as evidenced with the viability index of cells, which was 86 ± 4% and 98 ± 6% 48 h post-inoculation, when treated with 5 and 10 μM of FLX, respectively, whereas the value was below 20% when the infected cells were not treated. The infectious titer of supernatants was below the limit of detection 48 h after adding FLX to cell cultures, whereas it was (2.2 ± 1.6) × 10^6^ TCID_50_/mL in infected cells not treated with FLX (Figure 1b).

These data showing the antiviral effect of FLX in Vero E6 prompted us to study the effect of FLX in Vero E6 infected with SARS-CoV-2. No CPE was observed (data not shown), and the level of infectious particles in supernatants was below the limit of detection 24 h post-infection. However, 48 h post-infection, this level was 10^3.5^ TCID_50_/mL, as high as 10^5^ TCID_50_/mL the day after, and 2.3 × 10^6^ ± 1.8 × 10^6^ TCID_50_/mL 5 days post-infection. In comparison, the level of infectious particles in supernatants of untreated infected cultures was already over 10^5^ TCID_50_/mL 24 h post-infection and reached 2.3 × 10^6^ 72 h post-infection (Figure 1c).

In the experiments that followed, FLX (1, 5, or 10 μM) was added to Vero E6 cells cultures along with SARS-CoV-2, the cells were incubated for 2 h, the cells were washed, and the same concentration of FLX (1, 5, or 10 µM accordingly) was added before continuing with incubation.

When less than 10 µM of FLX was added to the cell cultures along with SARS-CoV-2, the infection was not inhibited. Indeed, 48 h post-infection, the viability indices of cells were 32 ± 6.5% and 20 ± 2% and infectious titers of supernatants were 2.4 ± 1.2 × 10^6^ TCID_50_/mL and 5.4 ± 3.9 × 10^6^ TCID_50_/mL when infected cells were treated with 1 and 5 μM of FLX, respectively. The same pattern of values was observed in untreated infected cells (Figure 1c). In contrast, in SARS-CoV-2-infected Vero E6 treated with 10 μM of FLX, the virus-induced cytopathic effect was inhibited. Indeed, 48 h post-infection, the cell viability index was higher in FLX-treated cells than in controls (83 ± 4% vs. 23 ± 7%; *p* = 0.01). Moreover, the infectious titers of supernatants were 2.1 ± 1.6 × 10^2^ TCID_50_/mL. In some experiments, the viral titer was below the limit of detection of the assay (Figure 1d).

**Figure 2:**
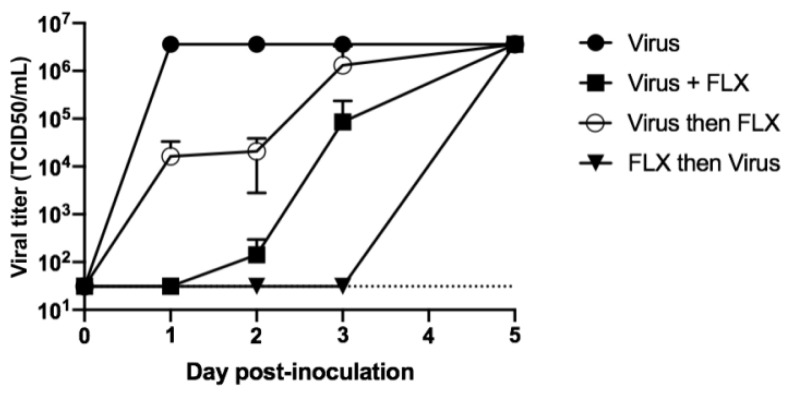
Levels of infectious particles in cell cultures treated with fluoxetine (FLX) before, during, or after inoculation of SARS-CoV-2. Vero E6 cells were seeded in 24-well plates at 10^5^ cells per well. The cells were inoculated with SARS-CoV-2 (black circles), SARS-CoV-2 + 10 μM of FLX (black squares), or SARS-CoV-2 incubated for 2 h and then treated with 10 μM of FLX (white circles), or the cells were incubated for 2 h in presence of 10 μM of FLX, followed by inoculation with SARS-CoV-2 and incubation for 2 h (black triangles). For each condition, the MOI was 0.01 and the cells were washed three times with complete medium after incubation and then treated again with 10 μM of FLX, followed by re-incubation. Levels of infectious particles in supernatants were determined using the endpoint dilution assay at days 1, 2, 3, 5 post-infection. The Spearman–Karber method was used to determine the tissue culture 50% infectious dose (TCID_50_/mL). The results are presented as the mean ± SD of three independent experiments.

In these experiments, it was observed that 10 μM of FLX could inhibit the infection of Vero E6 cells with SARS-CoV-2 when the molecule was added to the culture along with the virus and re-added after washings. Next, it was investigated whether an antiviral effect of FLX can be obtained when the molecule is added to cultures before or after the infection with SARS-CoV-2. To this end, Vero E6 cells were either inoculated with SARS-CoV-2 or with the virus and 10 μM of FLX or were treated with 10 μM of FLX for 2 h before or after inoculating the virus. In all experiments, cell cultures were washed with complete medium three times to eliminate any residual virus particle. Then, 10 μM of FLX was added or re-added according to the case for each condition except in controls. The level of infectious particles in supernatants was measured on days 1, 2, 3, and 5 post-infection. The level of infectious particles in supernatants of untreated cell cultures reached 3.16 × 10^6^ TCID_50_/mL as soon as 24 h post-infection. When the cells were inoculated with the virus along with FLX, the viral titer of supernatants of cell cultures was under the limit of detection 24 h post-infection, 1.42 ± 1.50 × 10^2^ TCID_50_/mL 48 h post-infection, and 3.16 × 10^6^ TCID_50_/mL 5 days post-infection. When the cells were infected and then treated with FLX 2 h post-infection, the levels of infectious particles in supernatants of cultures increased progressively and reached 1.3 ± 1.9 × 10^6^ TCID_50_/mL 3 days post-infection. When the cells were treated with FLX before the inoculation of SARS-CoV-2, the level of infectious particles in the supernatant of culture remained under the limit of detection up to 3 days post-infection and then reached 3.16 × 10^6^ TCID_50_/mL on day 5 post-infection (Figure 2).

Thus, the infection of Vero E6 cells can be inhibited when FLX is added to the cells before the inoculation of the virus and can be inhibited but at a lower extent and an extremely lower extent when FLX is added during and after the inoculation of the cells, respectively.

**Figure 3:**
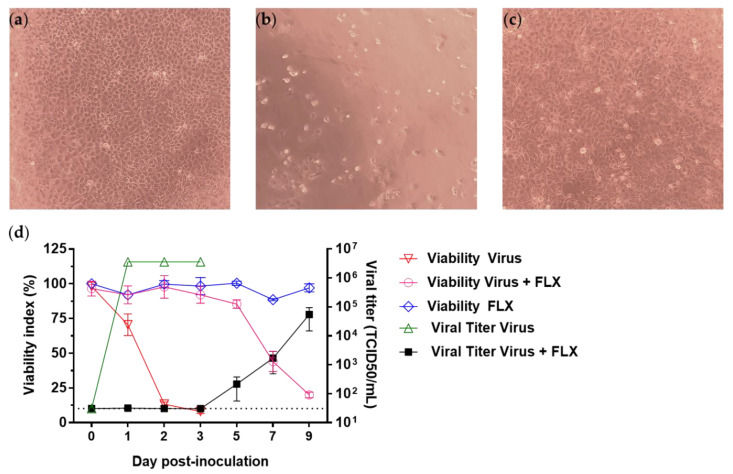
Levels of infectious particles in supernatants of SARS-CoV-2-infected Vero E6 cells treated daily with Fluoxetine (FLX). Cytopathic effects due to SARS-CoV-2 (MOI 0.01) in Vero E6 cells: (**a**) uninfected Vero E6 cells, (**b**) SARS-CoV-2-infected Vero E6 cells, (**c**) SARS-CoV-2-infected Vero E6 cells treated with 10 μM of FLX. The cells were observed under an inverted microscope (magnification ×100) after 72 h of incubation. (**d**) Vero E6 cells were inoculated with SARS-CoV-2 (MOI 0.01) and treated with 10 μM of FLX. After incubation for 2 h, the cells were washed three times with complete medium and then 10 μM of FLX was added. Cells were treated daily from day 1 to day 7 post-inoculation with 10 μM of FLX. Cell viability was evaluated using the Orangu assay (inverted gray triangles, white circles, dark gray diamonds) and levels of infectious particles in the supernatants were determined using the endpoint dilution assay (gray triangles and black squares) from day 1 through day 9 post-infection. The Spearman–Karber method was used to determine the tissue culture 50% infectious dose (TCID_50_/mL). The results are presented as the mean ± SD of three independent experiments.

To investigate further the effect of FLX on the infection of Vero E6 cells with SARS-CoV-2, cell cultures inoculated with FLX along with the virus were treated with 10 μM of FLX or DMSO on days 1, 2, 3, 4, 5, and 7 post-infection. While 100% CPE was observed in untreated infected cell cultures, no CPE was observed up to day 3 post-infection in FLX-treated cell cultures (Figure 3a–c). The effect of FLX on infected cells was evaluated using the Orangu viability assay and through the level of infectious particles in culture supernatants collected from day 1 through day 9 post-infection. The cellular viability of FLX-treated infected cells was around 100% on day 3 post-infection and 85% on day 5 post-infection, then the values were 44 ± 7% and 20 ± 2% on days 7 and 9 post-infection (Figure 3d), respectively. The viability of infected cells was 70 ± 7% on day 1 and 13 ± 1.5% as soon as day 2 post-infection (Figure 3d). The level of infectious particles in supernatants of FLX-treated cell cultures remained under the limit of detection until day 3 post-infection and was 2.17 ± 1.6 × 10^2^ TCID_50_/mL on day 5 post-infection and 5.4 ± 3.9 × 10^4^ TCID_50_/mL on day 9 post-infection. In contrast, in untreated infected cells, the level of infectious particles reached 3.6 × 10^6^ TCID_50_/mL as soon as day 2 post-infection (Figure 3d).

## 4. Discussion

We previously demonstrated that FLX inhibits CV-B4 in vitro and in vivo [25,26]. Hence, we decided to test the inhibitory effect of FLX on SARS-CoV-2 cultured in Vero E6 cells. The methodology of the current study is different in many respects from those of other teams. Indeed, FLX was administered after or before SARS-CoV-2 inoculation to cells, or simultaneously, followed by daily inoculation to evaluate the potential value of this molecule in the prophylaxis and/or the treatment of the infection. The infection of Vero E6 cells was evaluated through the CPE in Vero E6 cells line cultures, viability index compared with that of mock-infected cells, and level of infectious particles in supernatants of these cultures. The activity of FLX against SARS-CoV-2 was evaluated considering the inhibition of virus-induced CPE, cellular viability compared with that of mock-infected cells, and reduction of titers of infectious particles in supernatants.

It was determined that FLX was not cytotoxic in our system at concentrations lower than 50 μM and that FLX could inhibit the infection of Vero E6 cells with CV-B4. Interestingly, we observed that 10 μM of FLX inhibited the infection of these cells with SARS-CoV-2.

FLX can have a short half-life depending on the species [29], which can explain why administration of only one dose of the molecule to cultures resulted in the shifting of the onset of CPE and high levels of infectious particles in supernatants from day 1 to day 3 post-infection. It is also noteworthy that the patterns of results were different when cell cultures were treated with FLX before, during, or after the inoculation of SARS-CoV-2. The administration of FLX prior to the infection was more effective in delaying the onset of CPE and infectious particles in culture supernatants up to 3 days post-infection. A daily treatment of cultures with 10 μM of FLX also extended the delay. The effect of FLX observed in our experiments is reminiscent of the results of other studies reporting that 4.6 µM of FLX added to Vero cell cultures prior to the infection with SARS-CoV-2 at MOI 0.5 resulted in a reduction of the level of viral RNA in culture supernatants [20]. Our results regarding the effect of 10 µM of FLX added to cell cultures after the inoculation of SARS-CoV-2 are in agreement with those of Schloer et al. showing around a 2 log reduction of the viral titer in supernatants 48 h post inoculation [21].

**Figure 4:**
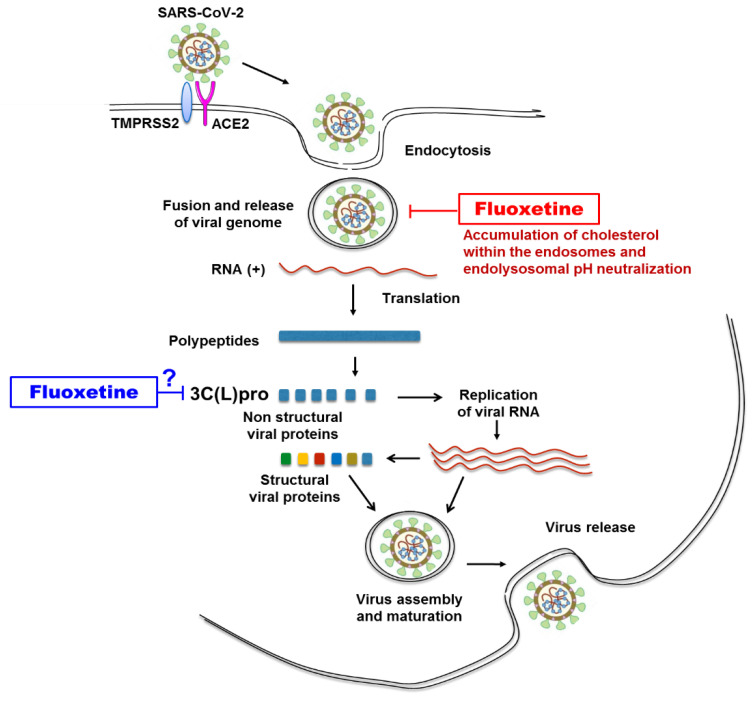
Antiviral activity of fluoxetine: known or potential targets of fluoxetine in steps of SARS-CoV-2 replication in permissive cell.

The FLX mechanisms involved in the inhibition of SARS-CoV-2 have been recently investigated. As far as enveloped viruses are concerned, they can enter cells through endocytic pathway and in this case the endosomal environment, including pH, proteases, ions, intracellular receptors, and lipid composition, is important to the virus–endosome fusion [30,31]. SARS-CoV-2 enters into host cell by the binding of its viral surface spike (S) protein to the angiotensin-converting enzyme 2 (ACE2) on the surface of the cell and the priming of the spike (S) protein by the transmembrane protease serine 2 (TMPRSS2), leading to virus particles endocytosis and fusion of the viral and cellular membranes [27] (Figure 4). Schloer et al. found that the treatment of Vero E6 cells with FLX can inhibit SARS-CoV-2 entry into cells through endocytic pathway by inducing accumulation of cholesterol within the endosomes [21] leading to inactivation of the endolysosomal proton pump responsible for pH maintenance and therefore resulting in impairment of endolysosomal acidification [32,33] (Figure 4).

FLX proved to be efficient in inhibiting several types of viruses through various other mechanisms. Regarding dengue virus, FLX most probably has an impact at the initiation of viral RNA replication [18], whereas in the case of CVBs, the putative target of FLX was the viral 2C protein whose function remains partially unknown [23,34].

Interestingly, similar to SARS-CoV-2, which contains a 3C-like protease, a target of SARS-CoV-2 drugs [35], coxsackieviruses contain a virally encoded chymotrypsin-like protease named 3C pro responsible for viral replication [36]. 3C-like proteases (3C(L)pro) are widely found in (+)ssRNA viruses. All of them are cysteine proteases with a chymotrypsin-like fold (PA clan), using a catalytic dyad or triad. They share some general similarities on substrate specificity and inhibitor effectiveness [37]. Whether FLX inhibits the replication of SARS-CoV-2 and CV-B4 through an effect on this protein deserves further study (Figure 4). FLX can inhibit SARS-CoV-2 and CV-B4 in Vero E6 cultures. The antiviral activity of FLX in these models opens a new avenue to study the interactions between these viruses and the cell and possibly to find new angles of attack. Elucidating the mechanism of the antiviral effect of FLX may pave way for novel combating strategies, especially for SARS-CoV-2.

FLX can also act on cytokine production and prevent cytokine storm in animal models of septic shock and allergic asthma through its anti-inflammatory activity [38,39]. Furthermore, in vivo experiments assessed FLX effects on inflammation in early brain injury, possibly involving the TLR4/MyD88/NF-κB signaling pathway [40]. Moreover, during SARS-CoV-2 infection, inflammation may have deleterious effects on the function of lungs and other organs and therefore on the survival of patients. Interestingly, it has been reported that the non-serotonergic anti-inflammatory effect of FLX potentially has the ability to inhibit IL-6 and NF-κB signaling pathways driving the cytokine storm in COVID-19 [41]. Taken altogether, these observations suggest that FLX could be relevant in vivo to control SARS-CoV-2 infection and to treat COVID-19. Recently the results of an in silico study suggested that the standard 20 mg, 40 mg, and 60 mg daily doses of FLX used in routine psychiatric treatments [42] achieve the non-toxic levels of FLX in lungs higher than 10 µM [43] which is a concentration effective to inhibit the infection with SARS-CoV-2 in vitro in our experiments and in those of others [21]. Thus, a COVID-19 therapy based on FLX might constitute a relevant approach to fight against SARS-CoV-2 infection and reduce COVID-19 severity. The results of in vitro or in silico studies are not an absolute guarantee of the effect in infected individuals; however, these encouraging observations deserve some attention and should prompt further studies to determine whether FLX has an effect on SARS-CoV-2 in primary cells and eventually in vivo.

In conclusion, FLX can inhibit SARS-CoV-2 in vitro. Interestingly, it was recently reported that 35 patients under treatment with a standard fluoxetine dose of 20 mg had a lower risk of intubation and death when they were hospitalized for COVID-19 [44]. It would be useful to assess the outcome of SARS-CoV-2 infection in a larger cohort of people receiving FLX, as it is a well-known treatment of depression worldwide. Further studies are needed to elucidate the mechanism of the antiviral effect of this molecule and investigate its potential in combating SARS-CoV-2 infections. Future studies will be directed along this line in our laboratory.

## Data Availability

Not applicable.
